# Relationship between the agonist activity of synthetic ligands of TRAIL-R2 and their cell surface binding modes

**DOI:** 10.18632/oncotarget.24526

**Published:** 2018-02-17

**Authors:** Neila Chekkat, Caterina M. Lombardo, Cendrine Seguin, Marie-Charlotte Lechner, Florent Dufour, Yves Nominé, Marcella De Giorgi, Benoit Frisch, Olivier Micheau, Gilles Guichard, Danièle Altschuh, Sylvie Fournel

**Affiliations:** ^1^ Laboratoire de Conception et Application de Molécules Bioactives, UMR 7199 CNRS, Université de Strasbourg, 67401 Illkirch, France; ^2^ Université de Bordeaux, CBMN, UMR 5248, Institut Européen de Chimie et Biologie, 33607 Pessac, France; ^3^ CNRS, CBMN, UMR 5248, 33600 Pessac, France; ^4^ Centre de Recherche Lipide, Nutrition et Cancer, UMR1231 Inserm, Université de Bourgogne Franche-Comté, UFR des Sciences de Santé, F21000 Dijon, France; ^5^ International Center of Frontier Research in Chemistry, F67083 Strasbourg, France; ^6^ Equipe labellisée Ligue 2015, UMR 7242, Université de Strasbourg, CNRS, ESBS, 67412 Illkirch, France; ^7^ Present address : Institut de Génétique et de Biologie Moléculaire et Cellulaire (IGBMC), INSERM U964, CNRS UMR 7104, Université de Strasbourg, 67404 Illkirch, France

**Keywords:** TRAIL-R2, signaling, interactions, binding modes, agonist peptides

## Abstract

Tumor Necrosis Factor Receptor Apoptosis Inducing Ligand (TRAIL) appears as an interesting candidate for targeted cancer therapy as it induces apoptosis in cancer cells without toxicity to normal cells. TRAIL elicits apoptosis through agonist death receptor TRAIL-R1 and TRAIL-R2 engagement. Nevertheless, recombinant soluble TRAIL and monoclonal antibodies against these receptors demonstrated insufficient efficacy in clinical trials. This may be explained by the cell-type dependency of the apoptotic response, itself influenced by the effect on ligand binding mode of factors such as the level of receptor oligomerization or glycosylation. To investigate the relation between binding mode and signaling, we used previously described synthetic divalent and monovalent peptides specific for TRAIL-R2. We measured their pro-apoptotic activity on three cancer cell lines sensitive to rhTRAIL induced-apoptosis and monitored their cell-surface binding kinetics. The two divalent peptides bound with strong affinity to TRAIL-R2 expressed on B lymphoma BJAB cells and induced a high degree of apoptosis. By contrast, the same peptides bound weakly to TRAIL-R2 expressed at the surface of the human colon cancer HCT116 or T lymphoma Jurkat cell lines and did not induce their apoptosis. Cross-linking experiments suggest that these differences could be afforded by variations in the TRAIL-R2 oligomerization state at cell surface before ligand addition. Moreover divalent peptides showed a different efficiency in BJAB apoptosis induction, and kinetic distribution analysis of the BJAB binding curves suggested subtle differences in binding mechanisms. Thus our data support a relation between the cell-surface binding mode of the peptides and their pro-apoptotic activity. In this case the precise characterization of ligand binding to the surface of living cells would be predictive of the therapeutic potential of TRAIL-R2 synthetic ligands prior to clinical trials.

## INTRODUCTION

Tumor Necrosis Factor Receptor Apoptosis Inducing Ligand (TRAIL, TNFSF10) is a type 2 transmembrane protein that binds to two agonist death receptors TRAIL-R1 (DR4, TNFR-SF10A) [1] and TRAIL-R2 (DR5, TNFR-SF10B) [2], as well as two decoy receptors TRAIL-R3 (DcR1, TNFR-SF10C) [3], and TRAIL-R4 (DcR2, TNFR-SF10D) [4]. TRAIL-R1/TRAIL-R2 receptors are type I transmembrane proteins that trigger apoptosis via their death domain in their intracellular region. TRAIL forms a homotrimeric ligand, that can be expressed at the membrane surface or cleaved by metalloproteases to form a soluble ligand. When bound to TRAIL-R1 or TRAIL-R2 at the cell surface, TRAIL enables the recruitment of the cytosolic protein FADD (Fas-Associated protein with Death Domain) to the receptors, which, in turn, recruit pro-caspase-8 [5]. The formed complex is called the Death Inducing Signaling Complex (DISC). After its activation in the DISC, caspase-8 cleaves and activates the executioner caspases leading to apoptosis through the extrinsic pathway. Depending on the cell type, caspase-8 can also cleave the pro-apoptotic protein Bid to induce the intrinsic pathway of apoptosis [6]. One important event in the TRAIL signaling pathway is the formation of a productive molecular complex between TRAIL and its agonist receptors at the membrane level [7]. Indeed, the valence and the affinity of this interaction are determinant to trigger an efficient apoptotic signal [8, 9]. Although the clustering of multiple TRAIL-R1 or TRAIL-R2 receptors induced by the trimeric ligand is not fully understood in terms of preorganization of the receptors prior to ligand binding [10, 11], of kinetics of the oligomerization process [12] and of the number of receptors required for optimal signal transduction [11, 13], it is commonly accepted that TRAIL-R2 requires a higher degree of oligomerization as compared to TRAIL-R1 [9].

TRAIL/TRAIL-Rs appear as promising targets for cancer therapy. Indeed TRAIL induces apoptosis in cancer cells while sparing the normal ones [14]. Thus, trimeric recombinant human TRAIL (rhTRAIL) and monoclonal antibodies (mAb) directed against TRAIL-R1 or TRAIL-R2 were developed for clinical treatment and demonstrated efficient anticancer activities in a number of preclinical studies [15]. Despite their promising potential, the first results from clinical trials are quite disappointing [16, 17], as TRAIL monotherapy generally did not induce cancer elimination. The assumption most often put forward is the acquired resistance to TRAIL-induced apoptosis of tumor cells [16]. Many mechanisms have been described, that can occur at any level of the TRAIL signaling pathway (reviewed in [18, 19]). A well-described mechanism involves the over-expression of anti-apoptotic molecules such as c-FLIP (Cellular FLICE (FADD-like IL-1β-converting enzyme)-inhibitory protein) as well as overexpression of proteins belonging to the Bcl-2 family or Inhibitory of Apoptosis Protein (IAP) family. Moreover, resistance can also occur at the cell membrane level, due to mutated TRAIL-R1/R2 receptors or because of the expression of the decoy receptors TRAIL-R3 or TRAIL-R4. It was first suggested that decoy receptors could be over-expressed on some cancer cell lines leading to apoptosis resistance. Although some studies confirm this hypothesis [20, 21], others emphasize the lack of correlation between decoy receptor expression and TRAIL-induced apoptosis resistance [22]. However, it was recently described that while TRAIL-R3 acts as competitor for the apoptotic receptors, the TRAIL-R4 decoy receptor can form heterodimers with TRAIL-R1 or TRAIL-R2 thus preventing apoptosis induction and leading to the activation of proliferative pathways such as NFκB or Akt [23, 24]. Finally, the mechanism of apoptosis resistance proposed to explain the poor efficiency of some mAbs, was an inefficient receptor clustering thus limiting DISC formation and apoptosis [25, 26]. Indeed, it was shown that some anti-TRAIL-R2 antibodies did not induce a sufficient degree of TRAIL-R2 oligomerization to trigger apoptosis and that the cross-linking of these antibodies was required to induce optimal apoptosis *in vitro* [9, 27]. To further investigate the mechanism of resistance, it seems crucial to characterize in detail the interaction between the various TRAIL-R2 binders and TRAIL-R2 at the membrane level.

In the present study, we investigated at the membrane level the cell dependent variability of the apoptosis induced by TRAIL-R2 specific ligands. For this purpose, we used synthetic multivalent peptides with a controlled degree of oligomerization that are specific of the TRAIL-R2 receptor (named TRAILmim/DR5), previously shown to induce TRAIL-R2-dependent apoptosis of BJAB cells when used as dimers or in higher oligomerization states [28]. Here we analyzed the ability for monomeric and dimeric peptides to induce apoptosis in three cancer cell lines, B lymphoma BJAB, T lymphoma Jurkat and colon cancer HCT116. We showed that while BJAB, Jurkat and HCT116 cells expressing TRAIL-R2 were all sensitive to the multivalent rhTRAIL, only BJAB cells underwent apoptosis after divalent TRAILmim/DR5 peptide treatment. To understand this discrepancy, we investigated the TRAIL-R2 binding properties of the peptides. We used surface plasmon resonance (SPR) to characterize their binding to recombinant TRAIL-R2 at a sensor surface, and the LigandTracer^®^ [29, 30] to monitor in real time their binding with TRAIL-R2 at the surface of living cells. Moreover we investigated the heterogeneity of kinetic data recorded with LigandTracer by kinetic distribution analysis [31] using the tool Interaction Map^®^ [32–34]. Our data suggest a relationship between the cell surface binding properties of the TRAIL-R2 ligands and their pro-apoptotic activity, which might be used as predictive tool of their therapeutic potential or that of monoclonal antibodies targeting TRAIL-R2 for clinical trials.

## RESULTS

### Divalent TRAILmim/DR5 induce apoptosis in BJAB cells but not in HCT116 and Jurkat cells

**Figure 1:**
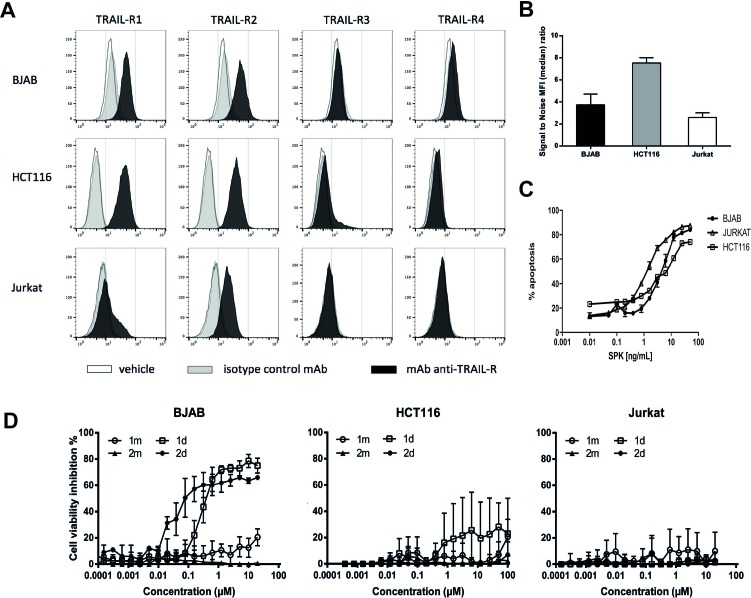
Divalent TRAILmim/DR5 induce apoptosis in BJAB cells but not in HCT116 and Jurkat cells **(A**, **B**) BJAB, HCT116 and Jurkat cells were stained with a monoclonal antibody targeting TRAIL-R1, R2, R3 or R4. The TRAIL receptor expression was monitored by flow cytometry. The resulting fluorescence histograms are showed in (A) and the signal to noise ratio of the median of fluorescence intensity between control isotype and TRAIL-R2 specific labeling, that are correlated with the level TRAIL-R2 expression, in (B). (**C**, **D**) Inhibition of cell viability, as deduced from metabolic activity measured by MTS assay for BJAB, HCT116 or Jurkat cells treated with different concentrations of SPK (C) or monovalent (1 m, 2 m) or divalent (1 d, 2 d) peptides (D). Results are expressed as % of cell viability inhibition according to the following formula: cell viability inhibition % = (OD treatment/OD (100% viability) ^*^ 100), were cells incubated with medium alone were considered as 100% of viability. Values correspond to the mean of 3 independent experiments ± SEM.

We previously described two cyclic peptides, named 1m and 2m in their monovalent forms that only differ by the position of a lysine in their sequence (see Supplementary Materials). Their divalent forms, known as 1d and 2d respectively, bound to TRAIL-R2 with high affinity as measured by SPR and induced apoptosis of various cell lines [27, 28]. In the present study, we compared the pro-apoptotic activity of 1d and 2d on the human Burkitt lymphoma BJAB, T leukemia Jurkat and the colon carcinoma HCT116 cell lines. As shown by flow cytometry using an anti-TRAIL-R2 antibody, these 3 cell lines express TRAIL-R2 (Figure 1A), with a similar amount for BJAB and Jurkat and twice lower than HCT116 (Figure 1B). BJAB and HCT116 express TRAIL-R1 but neither TRAIL-R3 nor -R4 (Figure 1A). As expected, the hexameric form of rhTRAIL named SPK (Figure 1C) induced apoptosis in the three cell lines. By contrast, while BJAB cells underwent apoptosis when treated with 1 d and 2 d (Figure 1D, left panel), two divalent TRAILmim/DR5 peptides, Jurkat or HCT116, albeit expressing TRAIL-R2, displayed strong resistance, and limited apoptosis only detected at the highest peptide concentrations (Figure 1D, middle and right panel). Noteworthy, 2 d was more efficient than 1 d in inducing BJAB cell death as shown by the IC_50_ of 0.03 µM for 2 d and 9 µM for 1 d.

### Divalent TRAILmim/DR5 peptides 1d and 2d bound strongly to the surface of BJAB cells expressing TRAIL-R2, but weakly to HCT116 and Jurkat cells

**Figure 2:**
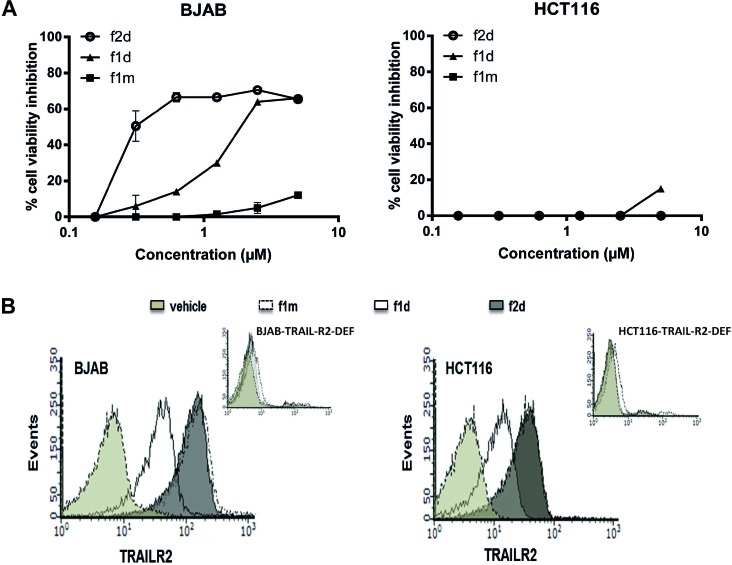
Fluorescent dye Atto488 does not affect peptide mono- or divalent binding activity on BJAB and HCT116 (**A**) MTS assay was assessed for BJAB and HCT116 cells treated with different concentrations of monovalent (f1 m) or divalent (f1 d, f2 d) peptides. (**B**) BJAB and HCT116 cells expressing or not TRAIL-R2 were stained with labeled peptides f1 d and f2 d and binding was monitored in flow cytometry.

To investigate the reasons why BJAB, Jurkat and HCT116 cells dysplay different sensitivity to 1d and 2d peptide-induced apoptosis, the binding of fluorescent (ATTO 488) 1 d (f1 d) and 2 d (f2 d) peptides onto living BJAB and HCT116 cells was monitored in real time using the LigandTracer technology (all experimental details related to the preparation of labeled peptides as well as analytical data can be found in the Supplementary data). In a first set of experiments, we verified that the presence of the fluorochrome did not affect the pro-apoptotic activity of the peptides (Figure 2A) or their capacity to interact with sensor immobilized recombinant TRAIL-R2 by SPR (see Table 1). Using flow cytometry, we also demonstrated that f1d and f2d bound on BJAB and HCT116 cells, but not on BJAB or HCT116 cells deficient for TRAIL-R2 (Figure 2B), indicating that the two fluorescent peptides bound on the cell surface in a receptor-dependent manner.

**Table 1:** Binding parameters for the interaction between the peptides and sensor-immobilized TRAIL-R2

Peptides	k_on_ (M^–1^ × s^–1^)	k_off_ (s^–1^)	K_D_ (M)
**1m**	(5.3 ± 1.5) × 10^3^	(2.6 ± 0.5) × 10^–3^	(5.1 ± 0.5) × 10^–7^
**1d**	(2.8 ± 0.2) × 10^4^	(1.4 ± 0.2) × 10^–3^	(5.1 ± 0.8) × 10^–8^
**2m**	(3.6 ± 2.8) × 10^3^	(1.6 ± 1.5) × 10^–2^	(3.6 ± 1.4) × 10^–6^
**2d**	(1.5 ± 0.3) × 10^6^	(1.8 ± 0.1) × 10^–4^	(1.3 ± 0.3) × 10^–10^
**f1m**	(1.4 ± 0.6) × 10^4^	(1.4 ± 2.9) × 10^–3^	(9.8 ± 1.8) × 10^–6^
**f1d**	(2.2 ± 3.5) × 10^4^	(1.4 ± 0.2) × 10^–3^	(6.6 ± 2.7) × 10^–8^
**f2d**	(1.5 ± 5.3) × 10^5^	(2.6 ± 0.7) × 10^–4^	(1.7 ± 0.7) × 10^–9^

**Figure 3:**
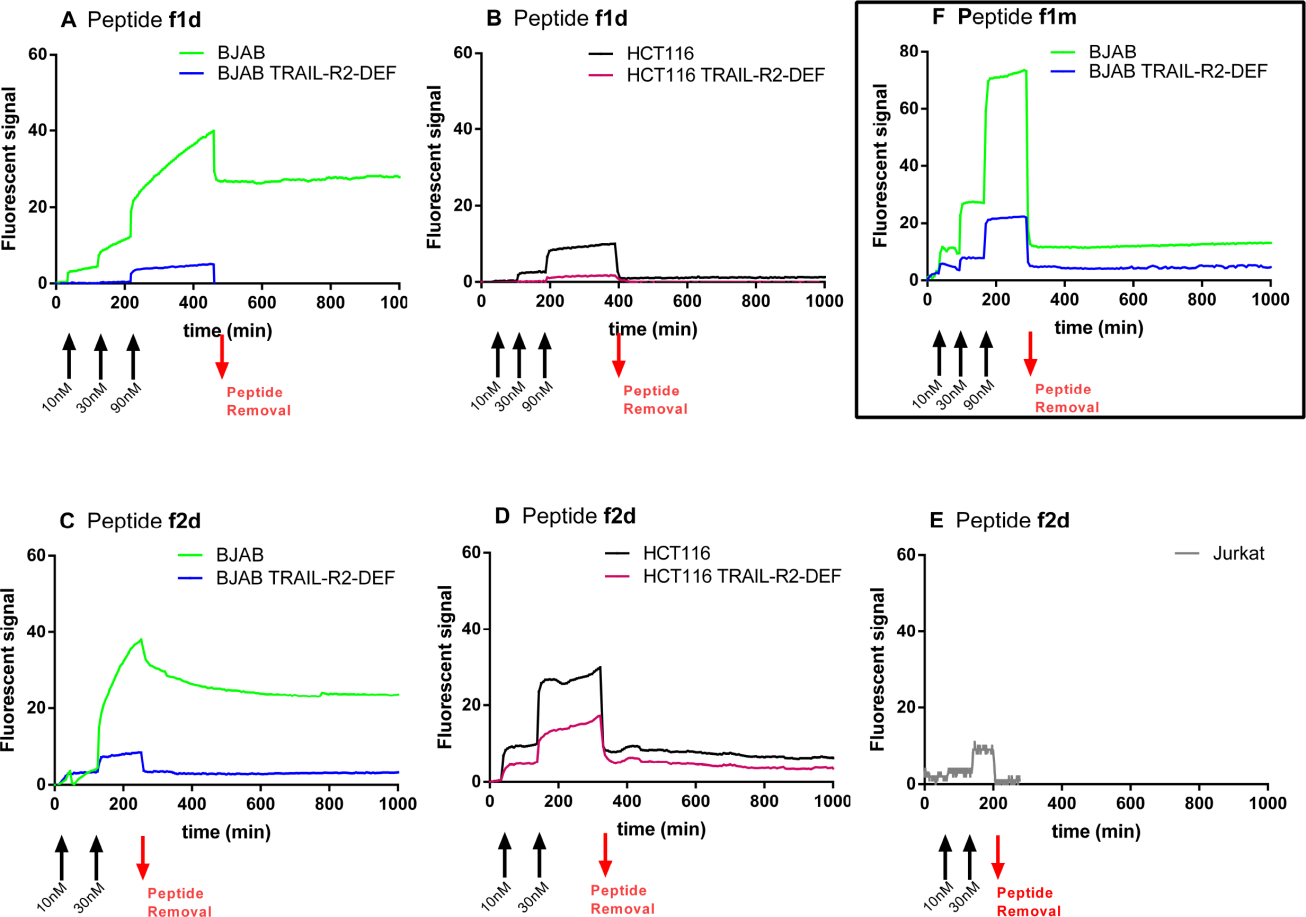
Divalent peptides f1d and f2d but not the monovalent f1m, form stable complexes with TRAIL-R2 expressed on BJAB cells whereas the same peptides bound weakly to HCT116 and Jurkat cells (**A–E**). Representative kinetic curves recorded with the LigandTracer for the interaction of divalent peptides f1d (A, B) and f2d (C, D, E) with (A, C): BJAB (green curves) and BJAB-TRAIL-R2-DEF (blue curves) cells or (B, D): HCT116 (black curves) and HCT116-TRAIL-R2-DEF (purple curves), or (E): Jurkat cells. (**F**) Binding curves recorded for the interaction between monovalent peptide f1m on BJAB expressing TRAIL-R2 (green curve) or not (blue curve). The black arrows indicate the addition of peptides at increasing concentrations (10 nM, 30 nM, 90 nM) as indicated. The red arrows indicate peptide removal.

The cell-surface binding kinetics of the dye-labeled peptides f1d and f2d were then analyzed with the LigandTracer. BJAB and HCT116 cells expressing or not TRAIL-R2 as well as the TRAIL-R2^+^ Jurkat cells were put in presence of increasing doses (10, 30 and, for f1d, 90 nM) of each fluorescent peptide, added successively approximately every two hours before removing the peptide to analyze the dissociation phase. Plots of the variation in fluorescence signal over time recorded with the peptides are shown in Figures 3. Despite some variations in fluorescence levels attributed to differences in the number of cells fixed onto the glass slide, fluorescent signals >25 were reached for both peptides f1d and f2d on BJAB cells that expressed TRAIL-R2 (Figure 3A and 3C, green curves). By contrast, the two fluorescent peptides produced weak signals when BJAB cells were deficient for TRAIL-R2 (Figure 3A and 3C, blue curves) showing that their binding was dependent on the presence of TRAIL-R2. The complexes between the peptides and TRAIL-R2 were quite stable, as they dissociated slowly after peptide removal from the solution. In contrast to their behavior with BJAB cells, the fluorescent peptides bound weakly to HCT116 cells expressing TRAIL-R2, and dissociation from these cells occurred nearly as rapidly after removal of the peptide (Figure 3B and 3D, black curve) as on HCT116 cells deficient for TRAIL-R2 (Figure 3B and 3D, purple curve). These observations suggest that the dimeric peptides bind to HCT116 cells that express TRAIL-R2, but with a weak complex stability. A weak binding signal, if any, was observed with the f2d peptide on Jurkat cells (Figure 3E). Weak binding was also observed when the fluorescent monovalent 1m peptide was incubated with BJAB cells (Figure 3E). Altogether these results indicate a relationship between the stability of the complexes (peptides/TRAIL-R2) formed at the cell surface and their pro-apoptotic activity: dimers f1d and f2d, that showed strong binding to BJAB cells, induced apoptosis whereas the same peptides bound weakly to Jurkat and HCT116 and scarcely induced their apoptosis. Similarly peptide f1m showed weak binding (Figure 3E) and did not induce apoptosis in BJAB cells (Figure 2A).

**Figure 4:**
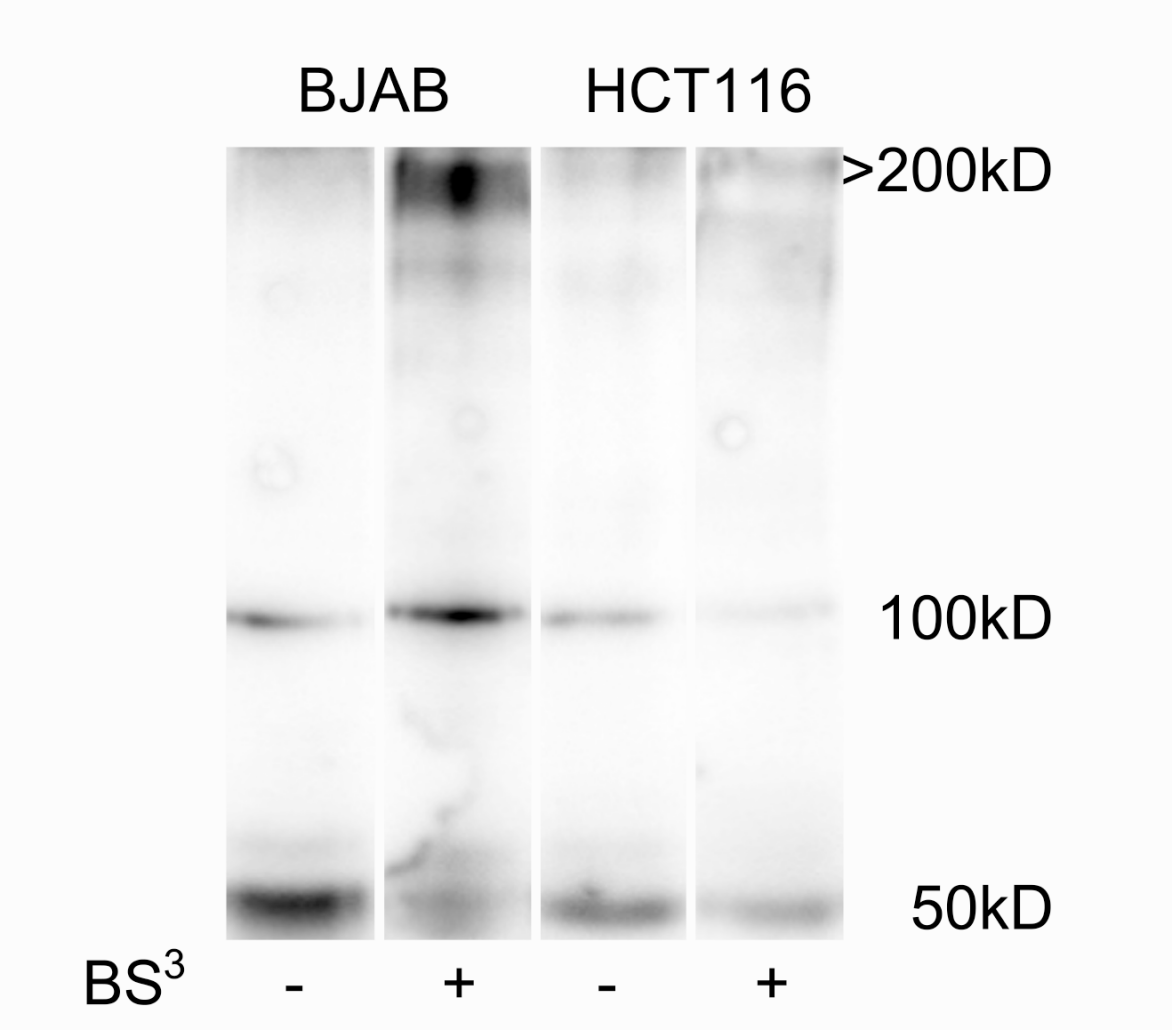
TRAIL-R2 is highly oligomerized before ligand addition in BJAB but not in HCT116 cells The two cell lines were incubated or not with 5 mM BS^3^ for 30 min. After, BS^3^ quenching and cell lysis, TRAIL-R2, monomer (50 kD) or crosslinked versions (>200 kDa), were revealed on western blot using a specific monoclonal anti-TRAIL-R2 antibody.

To go further in the molecular bases of these contrasted binding modes and ability to induce apoptosis, we investigated the oligomerization state of TRAIL-R2 at the cell membrane of the various cell lines in the absence of ligand. For this, HCT116 and BJAB were chemically cross-linked with the non-permeable reagent BS^3^ [35]. Western blot analysis revealed that a large portion of TRAIL-R2, migrating at ∼50 kDa, shifted to an oligomer of approximately 200 kDa in cross-linked cell lysates of BJAB (Figure 4). By contrast, the shift to the oligomeric form was nearly not occurring in HCT116 lysates. This discrepancy in TRAIL-R2 oligomerization state could explain the differences in binding mode and apoptosis induction observed with the divalent TRAILmim/DR5. Indeed the presence of oligomers at the cell surface could favor dimeric binding, resulting in complex stabilization.

### Divalent TRAILmim/DR5 1d and 2d present differences in their mode of binding to TRIAL-R2, both at sensor and at cell surfaces

Experiments showed in Figure 1D suggested that 2d was more efficient than 1d to induce BJAB apoptosis. To examine if this difference in efficiency might result from a difference in the mode of recognition, we analyzed the interaction of the dimeric peptides with sensor-immobilized recombinant TRAIL-R2 using SPR and further evaluated LigandTracer data recorded with TRAIL-R2 expressing BJAB cells (Figure 3A and 3C).

**Figure 5:**
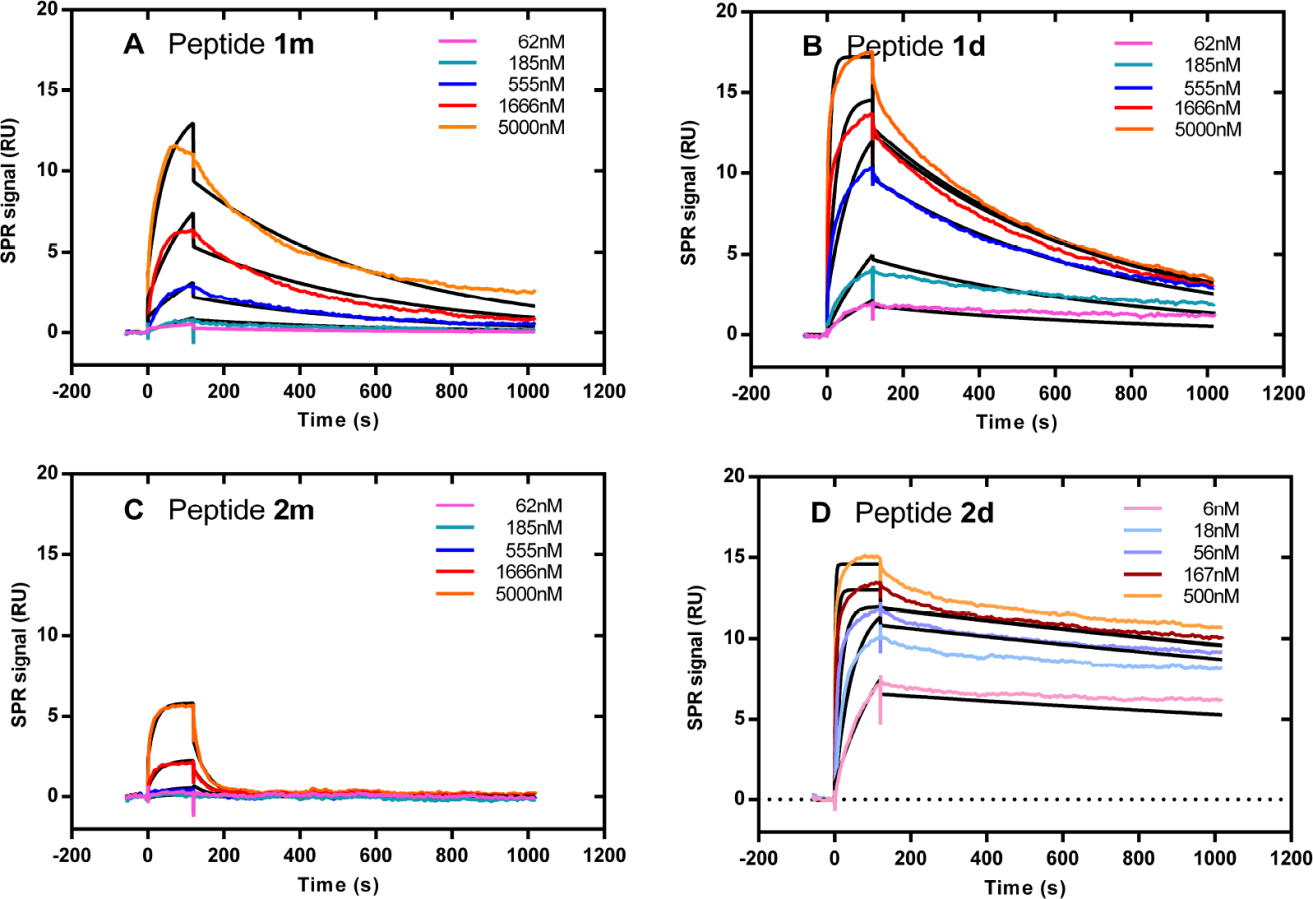
Divalent peptides 1d and 2d bind with stronger affinity than monovalent peptides 1m and 2m to sensor-immobilized TRAIL-R2 Representative kinetic SPR curves recorded with monovalent 1m (**A**) and 2m (**C**) and divalent 1d (**B**) and 2d (**D**) peptides. The fitted curves using the Langmuir one:one model are shown in black.

For this, the SPR binding profiles of the divalent (1 d and 2 d) and monovalent (1 m and 2 m) peptides injected on recombinant TRAIL-R2 immobilized on a sensor surface were compared (Figure 5). Kinetic parameters were calculated by global fitting of the kinetic curves using a Langmuir binding model. As shown in Table 1, the equilibrium constants (K_D_) calculated with the divalent peptides were always smaller than those of the monovalent peptides. Furthermore, the K_D_ obtained with peptide 2d was approximately 400 fold smaller than the K_D_ obtained with peptide 1d. This gain in affinity was due to a faster association (2d : k_on_ = 1.5 × 10^6^ M^–1^ s^–1^, 1d : k_on_ = 2.8 × 10^4^ M^–1^ s^–1^) and a slower dissociation (2d : k_off_ = 1.8 × 10^–4^ s^–1^, 1d k_off_ = 1.4 × 10^–3^ s^–1^). The dissociation rate constants recorded with the labeled peptides f1m, f1d and f2d were very similar to those recorded with the unlabeled peptides 1m, 1d and 2d, respectively (Table 1).

The stoichiometry of the interactions between divalent peptides and TRAIL-R2 was estimated from comparing the fitted R_max_ values (amount of peptide bound at surface saturation) of the divalent peptides 1d and 2d with that of the monovalent peptide 1m. If one divalent peptide molecule binds to one TRAIL-R2 molecule (monovalent binding), R_max_ calculated for the divalent peptide would be twice that of the monovalent peptide, because its molecular weight is twice that of the monovalent peptide. By contrast, if one divalent peptide molecule binds to two TRAIL-R2 molecules (bivalent binding), R_max_ would be the same for the monovalent and the divalent peptides. The R_max_ for 2m could not be fitted with confidence because the sample concentrations used were too low to approach surface saturation. As shown in Table 2, the R_max_ values for 1d and 2d were similar to that of 1m, suggesting that the divalent peptides prevalently bound in a “bivalent” way. As seen in Figure 5, the fits obtained with peptides 1d and 2d were not perfect. To further investigate the binding modes, we fitted the kinetic curves using complex interaction models instead of the Langmuir model. Although the use of more complex models implies an increase in the number of parameters to be fitted, and therefore a decrease in the resulting chi2 values (statistical parameter of the goodness of fit), the Chi^2^ values shown in Table 2 indicate that the bivalent analyte and the heterogeneous ligand models are significantly more appropriate to fit 2d data (∼10-fold smaller Chi^2^ compared to that obtained with the 1:1 model). On the contrary, the same complex models were not more appropriate than the Langmuir model to fit 1d data (similar Chi^2^ whatever the model is).

**Table 2:** Goodness of fit (Chi^2^) of two SPR data sets to simple and complex interaction models

			Model used for data fitting						
			Langmuir	Bivalent analyte		Heterogenous ligand		Two state reaction	
Peptide	Surface^a^	R_max_	Chi^2^	Chi^2^	Ratio^b^	Chi^2^	Ratio	Chi^2^	Ratio
**1 m**	1	33	0.92	0.88	1.0	0.59	1.6	0.76	1.2
	2	11	0.10	0.09	1.1	0.04	2.7	0.07	1.4
**1 d**	1	38	2.13	1.22	1.7	1.21	1.8	1.22	1.7
	2	13	0.25	0.15	1.7	0.15	1.6	0.15	1.7
**2 d**	1	33	4.54	0.49	9.3	0.32	14.4	2.04	2.2
	2	12	0.62	0.06	10.5	0.06	10.5	0.26	2.4

^a^Data obtained on two TRAIL-R2 surfaces with different R_*max*_ values, as fitted using the one:one interaction model, are presented.

^b^Ratio of the Chi^2^ obtained with the Langmuir model to the Chi^2^ obtained with the complex interaction models.

**Figure 6:**
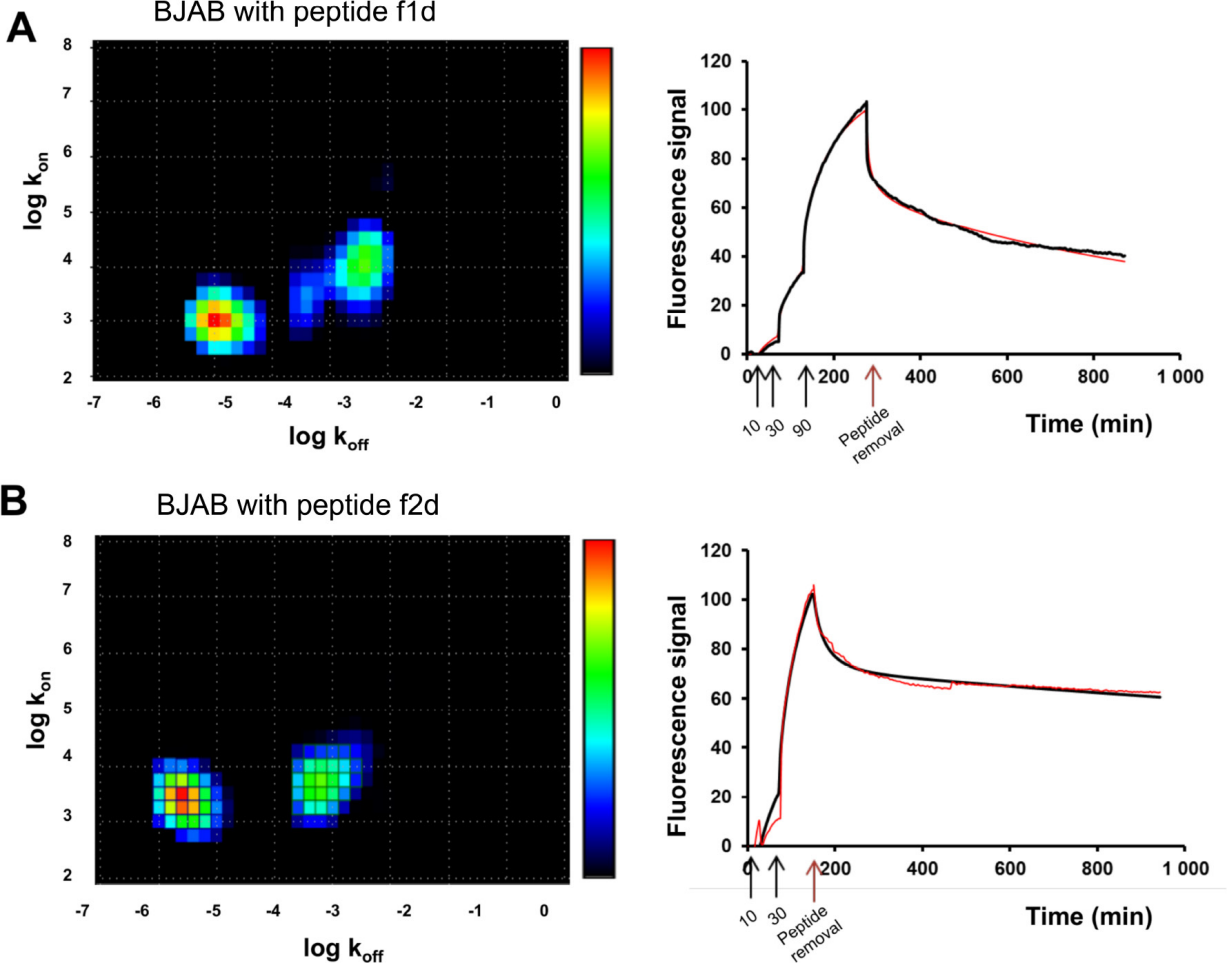
Divalent peptides f1d and f2d present differences in BJAB binding modes Representative interaction maps (left) calculated from real time LigandTracer binding curves (right, red: experimental binding curve, black: fitted curve) for the interaction between divalent peptides f1d (**A**) or f2d (**B**) on BJAB expressing TRAIL-R2. Colors represent the relative degree of contribution to the binding signal (red: large contribution, blue: small contribution). The black arrows on the curves (right) correspond to the addition of 10, 30 and 90 nM of peptide f1d (A) or 10 and 30 nM of peptide f2d (B) to the cells. The red arrows indicate the removal of peptides.

To go further in this analysis, the binding of f1d and f2d on BJAB cells, recorded with the LigandTracer (green curves in Figure 3A and 3C), were deciphered using a mathematical method (kinetic distribution analysis) that permits to investigate the heterogeneity of kinetic data. A visual inspection of the binding traces suggested differences in binding modes. Indeed a 90 nM concentration of f1d (Figure 3A) was required to reach sufficient curvature to perform kinetic fitting, while a 30 nM concentration of f2d was sufficient (Figure 3C). The fitted binding curves and corresponding Interaction Maps are shown in Figure 6. All maps show several peaks that differ predominantly by their position on the x-axis (stability), with a dominant high-affinity interaction (left peak). The residual binding signal was interpreted as a single low-affinity interaction (right peak) in 2d maps (Figure 6B) and as several ill-defined peaks in 1d maps (Figure 6A). Furthermore, the high-affinity peak tended to be positioned slightly higher on the y-axis (recognition) in 2d compared 1d maps, indicating a slightly faster association of 2d to the cell surfaces.

Overall, LigandTracer experiments and kinetic distribution analysis of the resulting data, which highlight subtle differences in the interaction of 1d and 2d with TRAIL-R2 expressed on BJAB living cells, are consistent with the kinetic differences in the TRAIL-R2 binding modes of 1d and 2d calculated from SPR data.

## DISCUSSION

Despite the detailed knowledge of signaling events downstream TRAIL-R activation, the initiating events in TRAIL-induced apoptosis, especially at the membrane level, are not clearly understood [7] probably because of the lack of tools that enable the monitoring of cellular binding [36, 37]. Indeed, biophysical methods such as SPR monitor the interaction of ligands with recombinant receptors immobilized on a surface, a set-up that only partially mimics receptor presentation at a cell surface. SPR binding data may not fully transpose to *in cellulo* conditions where, for example, receptor clustering is typically required for signaling. It is known that the TRAIL-R2 receptor is pre-oligomerized via its PLAD domain (Pre-Ligand Associated Domain) at the membrane surface in absence of TRAIL [10]. TRAIL binding on pre-oligomerized TRAIL-R2 leads to receptor clustering in a highly organized network that is required to induce efficient apoptosis [11]. At least five mAbs targeting TRAIL-R2 were developed and evaluated in clinical trials [15]. Unfortunately, most of them are weak agonists, most likely because they are unable to induce the receptor network configuration required for apoptosis. In these conditions, characterization of the binding of ligands targeting TRAIL-Rs is crucial to understand which properties are required to initiate apoptosis signaling and optimize TRAIL receptor-targeted strategies, and in particular for divalent agonists.

We previously described synthetic peptides (1 and 2) targeting the TRAIL-R2 receptor and that induce TRAIL-R2-dependent apoptosis of BJAB cells, in divalent and trivalent forms [27]. SPR experiments indicated that both 1d and 2d formed stable complexes with recombinant TRAIL-R2 covalently immobilized on a sensor surface. We show here that, while HCT116 and Jurkat cells were sensitive to hexameric rhTRAIL (SPK) induced apoptosis, the dimeric peptides largely failed to kill them. Results from flow cytometry experiment showed that the two cell lines express different amount of TRAIL-R2 receptors: HCT116 express much more receptors than BJAB or Jurkat but the two divalent peptides bound to TRAIL-R2 expressed on HCT116 and BJAB cell lines. Nevertheless, flow cytometry does not provide information on peptide binding properties, which might differ in the three cell lines and explain differences in apoptosis-induction efficiency. To investigate this point, we used the LigandTracer to monitor in real time the binding between the peptides and TRAIL-R2 receptors expressed on BJAB, Jurkat and HCT116 living cells. The LigandTracer technology, associated with the kinetic distribution analysis of the resulting binding traces, enabled a detailed comparison of the binding mode of the peptides to receptors on the two cell lines.

We succeeded in recording kinetic curves for the binding of the two dimeric and of one monomeric peptide (Figure 3) to three cell lines. In order to preserve physiological conditions, we used BJAB, HCT116 and Jurkat that express a natural level of TRAIL-R2 receptors and we demonstrated that binding was receptor-specific by monitoring peptide binding on cells that did not express TRAIL-R2. The binding signal observed in cells expressing TRAIL-R2 was systematically higher than that observed in cells deficient for TRAIL-R2, even though the increase was small in the case of HCT116 cells (Figure 3B and 3D). The faint signal observed in TRAIL-R2-deficient cells might result from unspecific binding due for example to the presence of the fluorescent dye.

The LigandTracer kinetic data reveal drastic differences in the binding modes to TRAIL-R2 depending on both the nature of the peptide and the cell type. The monomeric peptide dissociated more quickly from BJAB cells than the dimeric peptides. This behavior was also observed in SPR experiments using the recombinant receptor (Figure 5) and can be attributed to the avidity of the dimeric peptides for adjacent receptors on cell or sensor surfaces. Interestingly subtle differences were also observed between dimeric peptides in binding TRAIL-R2: the faster association of 2d compared to 1d was apparent in the maps calculated from LigandTracer data (Figure 6), although less striking than in SPR. The presence of two well-resolved peaks in the 2d maps may support the finding that SPR data were well fitted only with the bivalent or heterogeneous binding models.

In contrast to the peptide-dependence of binding, the cell-line dependence was not predictable from SPR data. The dimeric peptides, which both showed strong binding in SPR experiments, dissociated slowly from BJAB cells (Figure 3A and 3C), and immediately from HCT116 cells (Figure 3B and 3D). The cell-line dependence of binding has been repeatedly reported in cell-binding studies using the LigandTracer (for example [32, 33, 38–40]) but rarely related to biologic activity. We demonstrate here that the binding mode of the peptides to cell surfaces is correlated with their efficiency in inducing apoptosis as the divalent peptides formed stable complexes at BJAB cell surfaces and induced their apoptosis efficiently whereas the weak cell surface binding of the monovalent peptides on the two cell lines and of the bivalent peptides on HCT116 correlate with low induction of apoptosis. Besides the major differences in binding and apoptosis-induction between cell lines, we also observed subtle differences within the same cell line. Indeed the dimeric peptides 1d and 2d were not equivalent in their ability to induce apoptosis in BJAB cells (Figure 1D). The correlation between binding and apoptosis-induction may therefore hold true not only for overall affinity, but also for more subtle binding characteristics such as dissociation. In line with these findings, it is worthy to note that dissociation of rhTRAIL to TRAIL-R2 has been measured by SPR to be also pretty poor (O. Micheau, personal communication).

The molecular bases for these observed differences in binding mode and ability to induce apoptosis is still largely unknown. The divalent synthetic peptides are likely to discriminate differently between TRAIL-R2 derived structures or other TRAIL receptors, compared to rhTRAIL which induced apoptosis in BJAB, Jurkat and HCT116 cell lines. Features such as receptor nature, i.e. expression level, organization, location or glycosylation could explain why a dimeric peptide displays a lower binding and apoptosis efficiency, compared to the hexameric rhTRAIL in HCT116 cells. For example TRAIL-R2 might present a different pattern of O-glycosylation sites in BJAB, Jurkat and HCT116 cells thus facilitating or limiting the recruitment of receptors to induce apoptosis [41]. Another explanation could be the oligomerization state of TRAIL-R2 at the cell membrane. TRAIL-R2 on the cells can form homodimer leading to receptors clustering to a higher extend thus facilitating dimeric peptide binding and inducing a stronger apoptosis signal. We performed some experiments suggesting that this hypothesis should be privileged. Indeed, using cross-linking (Figure 4), we showed that TRAIL-R2 was expressed in a high oligomerization state in BJAB cells that displays a higher binding and apoptosis sensitivity in comparison with HCT116. TRAIL-R2 on the cells can form homodimer leading to receptors clustering to a higher extend thus facilitating dimeric peptide binding and inducing a stronger apoptosis signal. TRAIL-R2 can also form heteromers with the inhibitor receptor TRAIL-R4 as described in publications [23, 24] thus limiting the TRAIL-R2 free receptors available to initiate apoptosis. By contrast, this hypothesis seems more unlikely as we demonstrated that TRAIL-R4 is not expressed on the cell surface of the three cell line used in this study (Figure 1A). We also could exclude an effect of TRAIL-R2 internalization as we previously showed (27) that 2d peptide induce a significant amount of internalization in the three cell lines.

In conclusion, we demonstrate that, while divalent peptides 1d and 2d bound TRAIL-R2 with strong affinity in SPR experiments, their binding mode at the cell surface, as well as their efficiency in cell apoptosis-induction, was cell-line dependent. Based on the observed correlation between peptide binding/dissociation at cell surfaces and pro-apoptotic activity, we propose that the detailed characterization of ligand-cell binding kinetics could be developed as a predictive tool to gain information about the therapeutic efficiency of new divalent ligands but also to rationalize the outcome of cellular assays. For example, it would be tempting to use the LigandTracer to determine the kinetic profiles on cells of a series of known mAbs selected for their high affinity for TRAIL-R2 and agonistic potential, and to correlate the results with their cellular activity profiles.

## MATERIALS AND METHODS

### Peptide synthesis

TRAILmin/DR5 peptides 1m, 2m, 1d and 2d were synthesized in our laboratory according to previous reports [27, 42]. The synthesis of the corresponding fluorescent versions namely 1m-Alexa 488 conjugate (f1m), 1d-ATTO 488 conjugate (f1d) and 2d-ATTO 488 conjugate (f2d) is describe in detail in supporting information. The formulae of all ligands are provided in the Supplementary data. Peptides 1 m, 2 m, 1 d and 2 d were solubilized in H_***2***_O and prepared in a stock solution at 5 mM. Fluorescent conjugate peptides were solubilized in DMSO in a stock solution at 100 µM.

### Cell lines

The human T leukemia Jurkat cells provided by ATCC (ATCC number TIB-152) were described to lack TRAIL-R1, R3 and R4 (Figure 1A).

The human Burkitt lymphoma BJAB cells deficient for TRAIL-R2 (BJAB-TRAIL-R2-DEF) as well as BJAB cells in which the expression of TRAIL-R2 was recovered by stable expression of TRAIL-R2 were obtained as described [43] and kindly provided by Andrew Thorburn (Department of Pharmacology, University of Colorado Denver School of Medicine, USA). HCT116 human colon cancer cell lines (HCT116 and HCT116-TRAIL-R2-DEF) that differ in the presence or absence of TRAIL-R2 expression at cell membrane were obtained after transfection using a pair TRAIL-R2 TALEN plasmids targeting exon 3 [44]. Both cell lines and isogenic derivatives were cultured in RPMI 1640 supplemented with 10% FBS (Fetal Bovine Serum), 100 U/mL penicillin and 0.1 mg/mL streptomycin. Puromycin antibiotic (0.5 µg/mL) was added to the BJAB medium to ensure the maintaining of TRAIL-R2 expression. Cells were maintained at 37° C with 5% CO2.

### Cell viability assay

BJAB, Jurkat and HCT116 cells (10^5^ cells/well) were cultured in 100 µL of culture medium in 96-flat bottom well plates. The next day, cells were treated with the indicated concentrations of the different TRAILmin/DR5 peptides. After 24 hours of treatment cell viability measurement was performed by the MTS assay according to the manufacturer specification (Promega Corporation, Madison, WI, USA).

### Crosslinking and western blot analysis

TRAIL-R2 oligomerization state was evaluated by a chemical cross linking followed by a classical western blot assay as previously described by our group [32]. Briefly, Bis(sulfosuccinimidyl) suberate (BS^3^) is an homobifonctionnal, amine reactive, non cleavable cross-linker with an 11.4 Å spacer arm, synthetized in our laboratory. BJAB and HCT116 cells were washed once in PBS, crosslinked with 5 mM BS^3^ for 30 min at room temperature then quenched with 20 mM glycine 15 min. Cells were pelleted by centrifugation and lysed in RIPA buffer 1h on ice. Total protein concentrations were determined by BiCinchoninic Acid protein assay and 20 µg were loaded on an 8% SDS polyacrylamide gel. Proteins were transferred on a PVDF membrane and probed using an anti-TRAIL-R2 (Millipore).

### Staining for flow cytometry analysis

10^6^ cells were washed in PBS containing 2% FCS and then incubated at 4° C for 30 min with an anti-TRAIL-R2 antibody (clone BK9 Diaclone, Besançon, France) according to concentration recommended by the manufacturer or with fluorescent peptides at the concentration of 1 µM. After two washes in PBS-2% FBS, TRAIL-R2 expression was monitored by flow cytometry (Guava easyCyte™, Merck Milipore, Darmstadt, Germany) and data were analyzed with InCyte Software (Merck Milipore).

### Surface plasmon resonance

SPR assays were performed on a Biacore T200^™^, at 25° C. The running buffer was HBS-EP [10 mM HEPES (pH 7.4) containing 0.15 M NaCl, 3.4 mM EDTA and 0.005% (v/v) Tween P20]. The human TRAIL-R2 (Enzo Life Sciences, Farmingdale, NY, USA) receptor was immobilized on a sCM5 sensor chip (GE Healthcare) using the standard amine coupling procedure. The receptors were diluted in 10 mM acetate buffer (pH 5.0) at a concentration of 5 μg/mL. The TRAIL-R2 immobilization level on the sensor chip was 430 RU. Peptides were injected at a flow rate of 5 μL/min for 120 s and allowed to dissociate for an additional 420 s. Surfaces were then regenerated for 5 s with 25 mM HCl. All binding curves were double-referenced (i.e. subtraction of the data of the empty flow cell followed by the subtraction of the data from a running buffer injection cycle).

### Real time kinetic measurement assay on living cells

The adherent cells HCT116-TRAIL-R2-DEF and HCT116 cells were directly plated on glass slides (Ø20 mm) (10^6^ cells/well). The cells in suspension (BJAB and BJAB-TRAIL-R2-DEF) were covalently adhered to glass slides coated with poly-L-lysine (0.1% v/v) (Sigma Aldrich, Saint Louis, MO, USA). The adhesion was strong enough for measurement during 10h. After 24 hours, the slides were fixed with superglue at different locations of a Petri dish, which was inserted in the LigandTracer Green instrument [28, 29] (Ridgeview Instrument AB, Uppsala, Sweden). An empty spot or empty glass slide fixed to the Petri dish was used to record the background signal. This set-up allowed the simultaneous monitoring of the binding of a given peptide to different cell-carrying glass slides fixed to the dish. Three mL of solution (culture medium without or with fluorescent peptide) were added to the dish. In the LigandTracer instrument, the dish is tilted and rotates. Consequently the 3 mL solution covers only the lower part of the dish and each cell-carrying slide is alternatively in contact and out of contact with the solution (at intervals of 8 to 16 s). The fluorescence signal is recorded over time when the slide is out of the solution, and therefore corresponds to the amount of bound peptide. The solution in the dish was initially the culture medium (∼30 min) to provide a stable base line. The fluorescent peptide was then added in increasing doses (10, 30 and 90 nM) every two to three hours. Finally the peptide-containing sample was replaced with fresh medium and the dissociation of the peptide from the cells was followed overnight. The measurements were conducted at room temperature (approximately 20° C).

### Evaluation of kinetic data

Double-referenced SPR binding curves sets were fitted globally using the BIAevaluation 4.1.1 (GE Healthcare Biacore) or TraceDrawer (Ridgeview Diagnostics AB, Uppsala, Sweden) softwares. The signal recorded with the LigandTracer on an empty spot or slide was subtracted from that recorded on cell-carrying slides. The resulting binding curves were analyzed by kinetic distribution analysis [31] using the tool Interaction Map (Ridgeview Diagnostics AB, Uppsala, Sweden) [32, 33]. Interaction Map decomposes the kinetic curve recorded for the binding of a homogeneous ligand to a heterogeneous group of targets into its different components. Results are represented as interaction maps (k_on_ versus k_off_, log scale), where the color of each populated area represents its degree of contribution to the kinetic curve (red = high contribution, blue = low contribution).

### Statistical analysis

Results from cell viability experiments were expressed as the mean ± SEM. The statistical significance was evaluated by a non-parametric ANOVA 2 way test plus a Bonferonni correction using GrapPad Prism 5.0 software. Results with a *p*-value less than 0.05 were considered significant.

## SUPPLEMENTARY MATERIALS


