# Supplementary material for: Relationship between the agonist activity of synthetic ligands of TRAIL-R2 and their cell surface binding modes
Source: Oncotarget. 2018 Feb 17;9(21):15566–78. doi: 10.18632/oncotarget.24526 (PMC5884648; doi:10.18632/oncotarget.24526)
Supplement: Supplementary file 1 [file oncotarget-09-15566-s001.pdf]

Relationship between the agonist activity of synthetic ligands of TRAIL-R2 and their cell surface binding modes

SUPPLEMENTARY MATERIALS

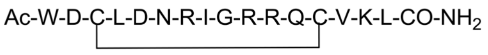

1m

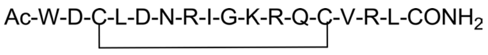

2m

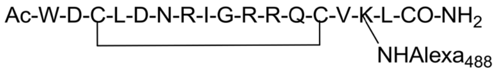

f1m

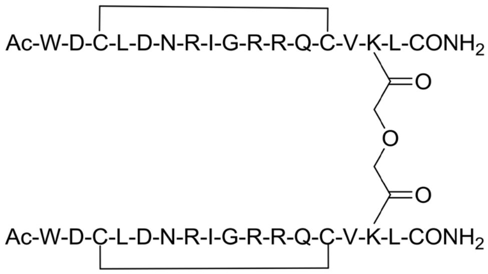

1d

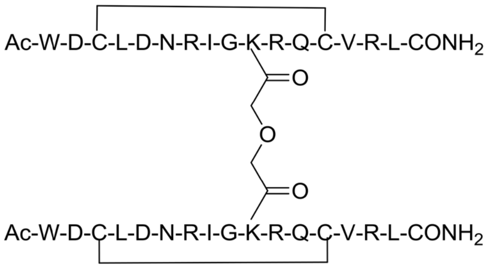

2d

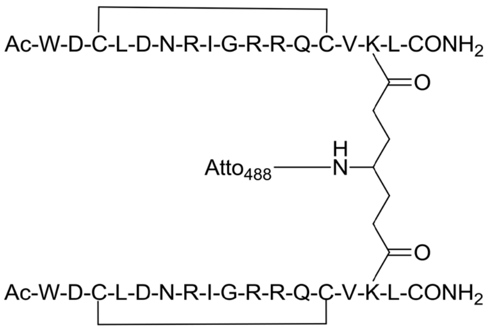

f1d

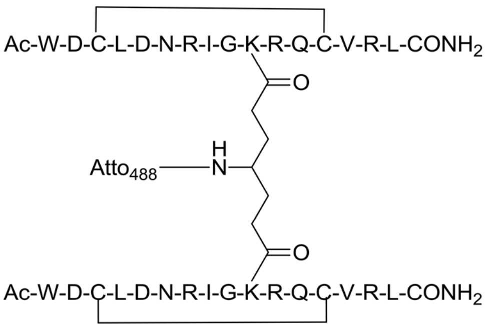

f2d

CHEMISTRY EXPERIMENTAL

General

Resin, protected amino acids and coupling reagents were purchased from PolyPeptide Group (Strasbourg, France) and Iris Biotech (Marktredwitz, Germany). Chemical reagents were purchased from Sigma–Aldrich and Alfa-Aesar and peptide grade solvents were purchased from Carlo Erba. Dyes Atto 488 and Alexa Fluor® 488 were respectively purchased from AttoTech and ThermoFiscer Scientific. Analytic HPLC characterisations were performed on a Dionex Ultimate 3000 system, using a Macherey-Nagel column (Nucleodur cc 70/4 100-3 C18 ec, 4.6 × 100, solvents acetonitrile/water 0.1% TFA,

1 ml/min). Semipreparative HPLCwas performed on a Dionex Ultimate 3000 system, using a Macherey-Nagel column (Nucleodur 100-16 C18 ec, 10 × 250, solvents acetonitrile/water 0.1% TFA, 4 ml/min). Preparative HPLC was performed on a Gilson Preparative HPLC system using a Macherey-Nagel column (Nucleodur C_{18} column 20 × 250 mm,solvents acetonitrile/ water 0.1% TFA, 20 ml/min) and UV detection at 220 nm. Low resolution ESI mass spectra were recorded with an LCQ Advantage/LC Surveyor apparatus (Thermo Finnigan). High resolution ESI mass spectra were performed on a Thermo Exactive apparatus.

Synthesis of dye conjugated compound f1m

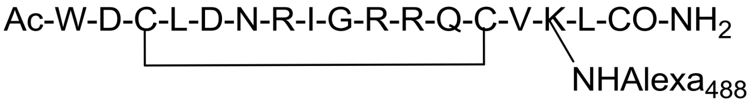

f1m

Peptide 1 m (3.2 mg, 1.3 µmol, synthesis previously reported)[1] was dissolved in 50 µl of DMF. Alexa488-NHS (1 mg, 1.5 µmol) was added to the peptide as a DMF (100 µl). DIEA (initially 0.2 µl) was added to the reaction to reach pH = 9, and the reaction was stirred at room temperature, monitoring with HPLC and maintaining the pH basic. The reaction terminated after 3 h, with an overall addition of 4 eq of DIEA. The reaction was quenched with 700 µl of H_2O and the crude was purified with semipreparative HPLC (20–50% acetonitrile 0.1% TFA, 30 min), and the desired product was obtained as a red solid after lyophilisation (2.1 mg, yield 64%). MW: 2531.7804; Exact mass: 2529.9923; Analytic HPLC (20%–50% CH_3CN 0.1% TFA, 10 min) rt 5.3 min, purity >99%; HRMS: *m/z measured* 2530.0466 (1266.0233 $[M+2H]^{2+}$, 844.3516 $[M+3H]^{3+}$).

HPLC

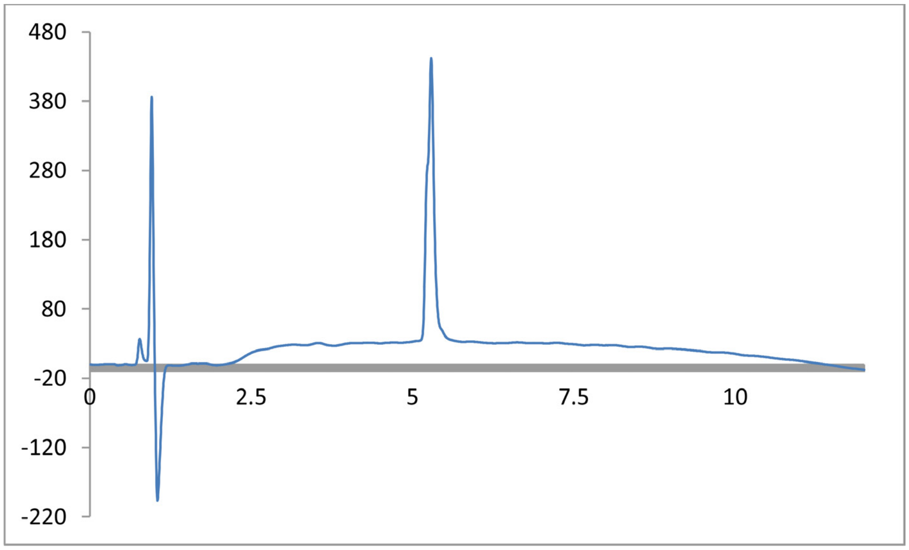

ESI

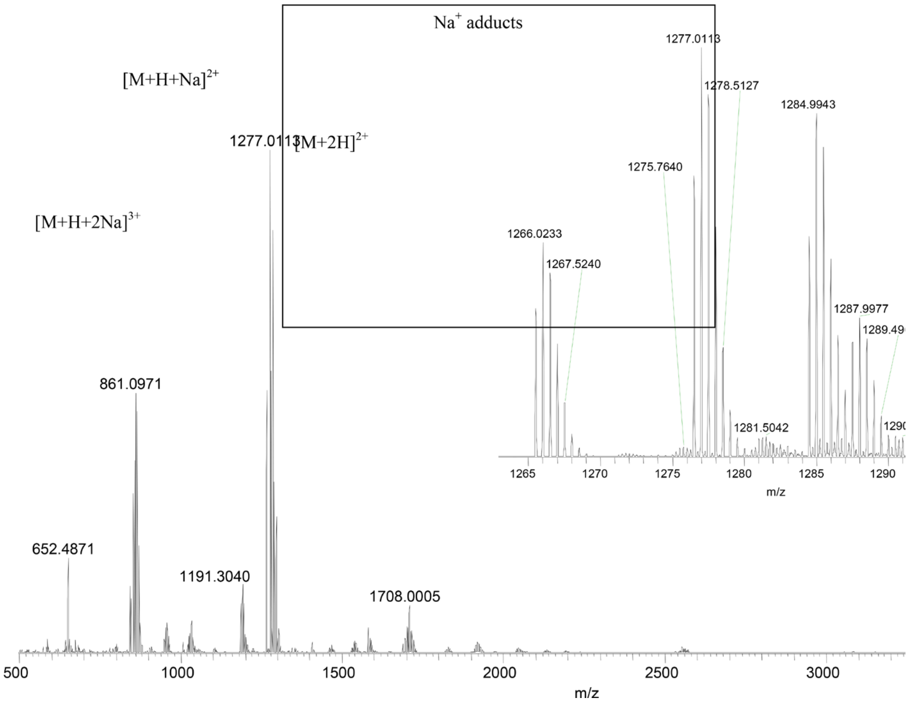

Synthesis of compound (1)

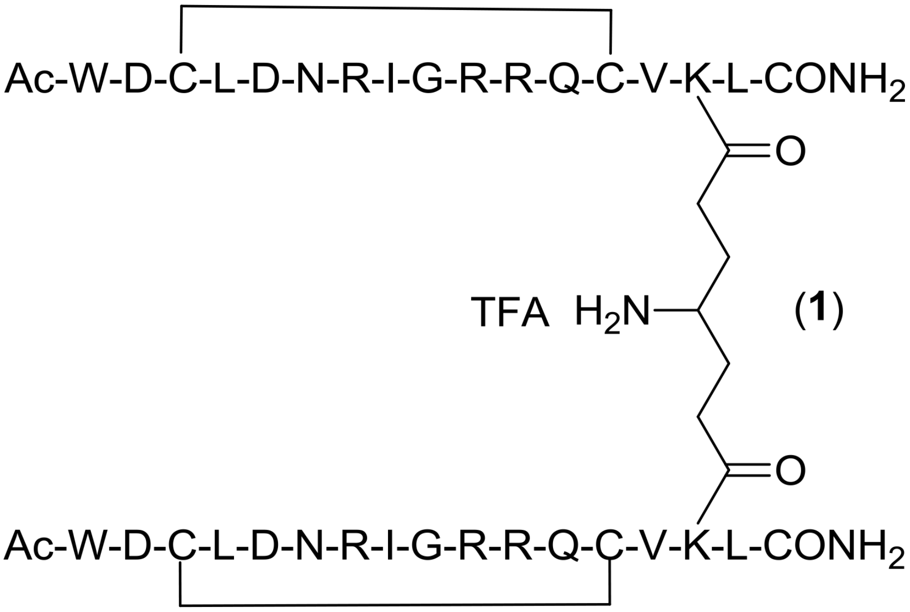

(1)

The compound was synthesised following the procedure previously reported for m2.

[2]Starting from 5.9 mg of protected dimer (1.38 µmol), the TFA salt of the desired compound was obtained as an off white solid (7.2 mg, quantitative). MW: 4283.82 (TFA salt); Exact mass: 4167.11; Analytic HPLC: Rt 5.6 min (20%–50% CH_3CN 0.1% TFA, 10 min), purity 98%; ESI-MS: (low res): *m/z measured* 1389.81 $[M+3H]^{3+}$, 1042.89 $[M+4H]^{4+}$, 834.55 $[M+5H]^{5+}$, 695.72 $[M+6H]^{6+}$, 596.47 $[M+7H]^{7+}$.

HPLC crude

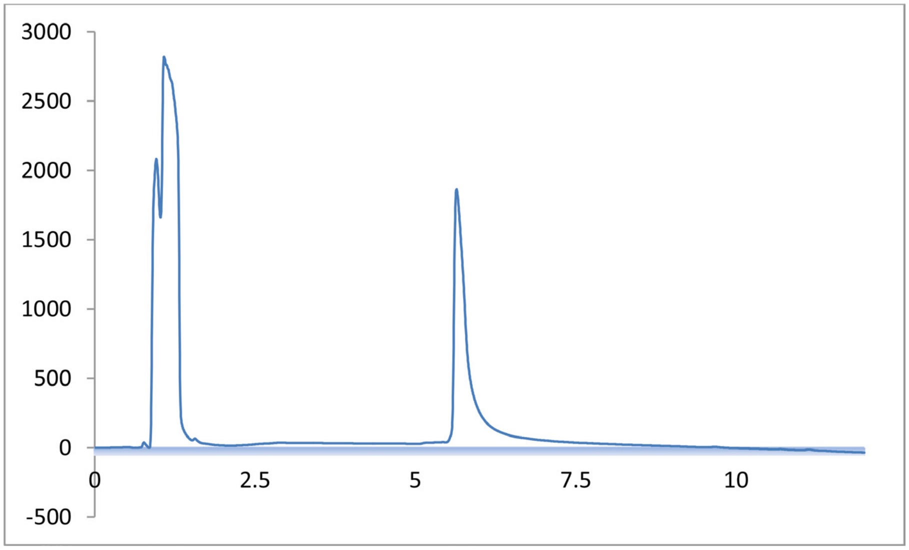

ESI-MS

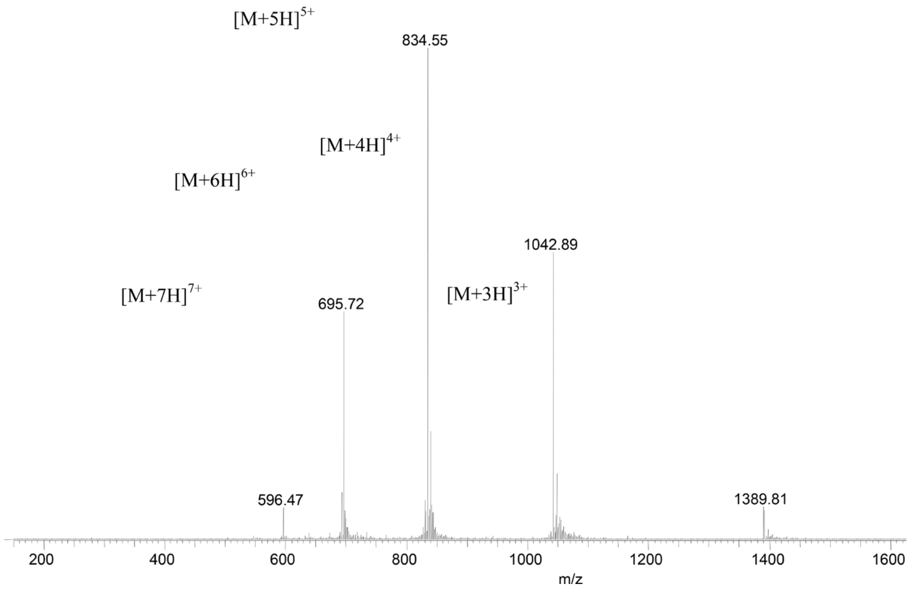

Synthesis of compound (2)

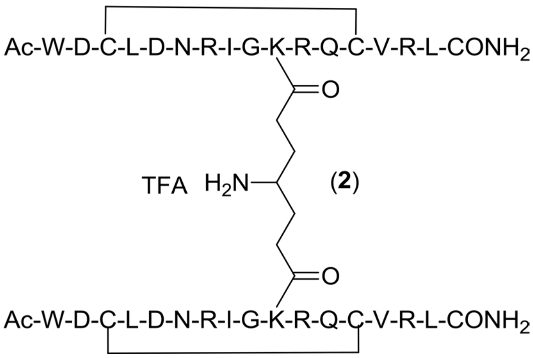

(**2**)

The synthesis of compound (2) has been previously reported[1].

Synthesis of dye conjugated compound f1d

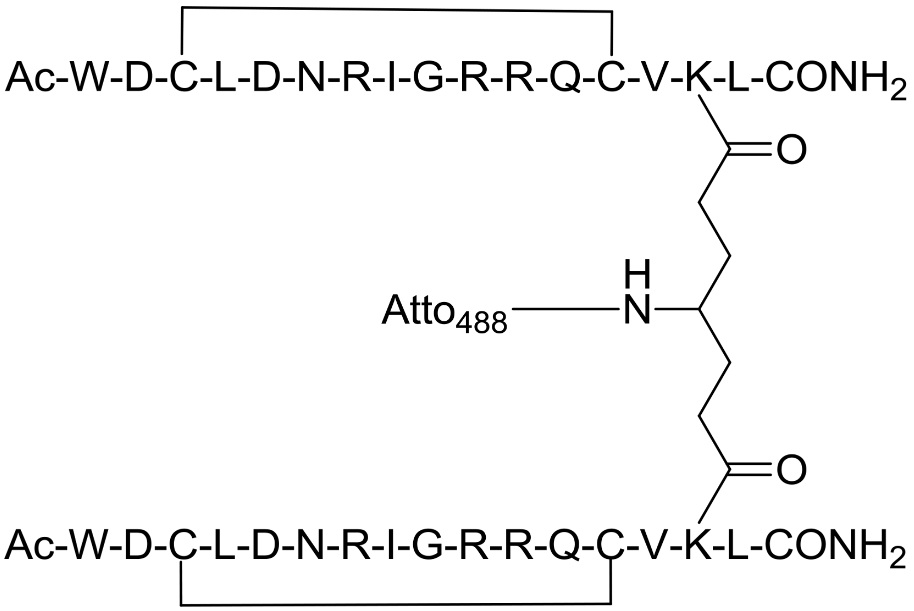

f1d

Compound (1) (2.6 mg, 0.52 µmol MW: 4965, considering 7 × TFA salt) was dissolved in 100 µl of DMF and stirred at room temperature. ATTO-488-OSu (0.6 mg, 0.63 µmol) was dissolved in 100 µl of DMF and added to the reaction, rinsing with DMF (100 µl). DIEA was subsequently added (initially 0.3 µl, 2.0 µmol, as a 1% solution in DMF) to reach a pH of 8–9, and the reaction was stirred at room temperature, monitoring the pH and the progression with HPLC (20%–50% CH_3CN 0.1% TFA, 10 min). The reaction terminated after 21 h, with an overall addition of 11 eq of DIEA. The reaction was quenched by addition of H_2O until neutral pH (300 µl), and the crude product was purified with semipreparative HPLC (gradient 20%–50% CH_3CN 0.1% TFA, 30 min, 220 nm), to afford the desired compound as an orange solid (1.5 mg, 52%). MW: 4737.6612 (neutral); Analytic HPLC (20%–50% CH_3CN 0.1% TFA, 10 min) rt 6.73 min, purity > 99%; HRMS: *m/z measured* 4737.2159 (1580.4053 $[M+4H]^{3+}$, 1185.5582 $[M+5H]^{4+}$, 948.8481 $[M+6H]^{5+}$, 794.5370 $[M+7H]^{6+}$).

HPLC

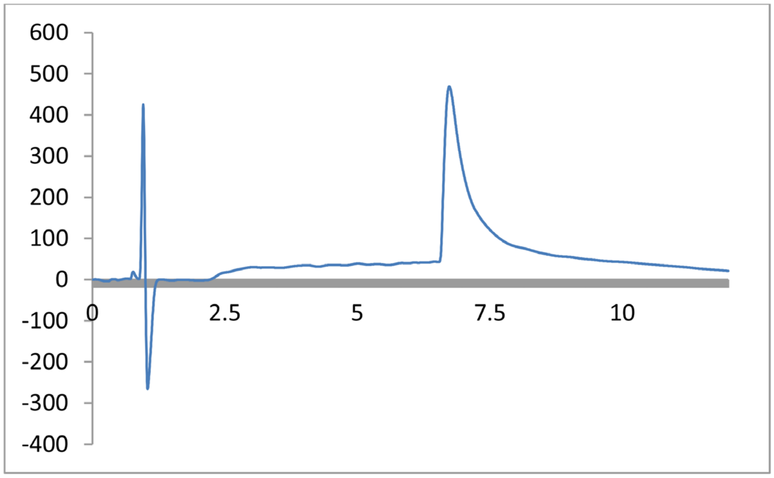
600
500
400
300
200
100
0
-100
-200
-300
-400
0
2.5
5
7.5
10

ESI

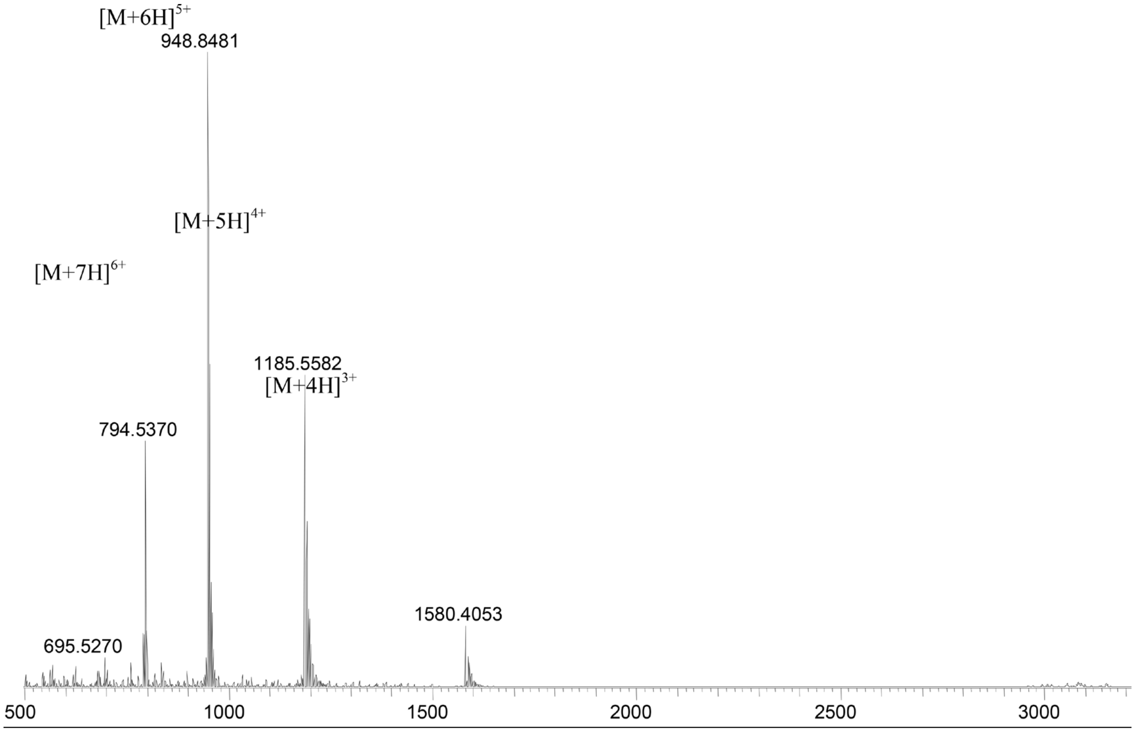
[M+6H]5+
948.8481
[M+5H]4+
[M+7H]6+
1185.5582
[M+4H]3+
794.5370
1580.4053
695.5270
500
1000
1500
2000
2500
3000

Synthesis of fd2

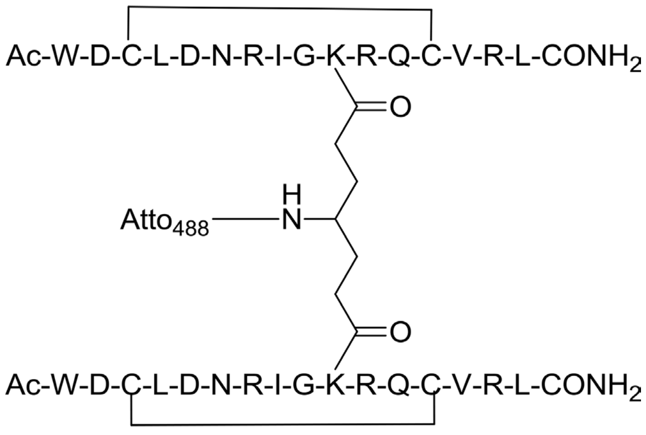

f2d

Compound (2) (6.5 mg, 1.6 µmol, MW: 4965, considering 7 × TFA salt) was dissolved in 250 µl of DMF and added to solid ATTO-488-OSu (1.6 mg, 2.0 µmol), stirring at room temperature. DIEA was subsequently added (initially 1.4 µl, 7.8 µmol) to reach a pH of 8–9, the reactor was rinsed with 350 µl of DMF and the reaction was stirred at room temperature, monitoring the progression with HPLC (20%–50% CH_3CN 0.1% TFA, 10 min) and the pH. The reaction terminated after 15 h, with an overall addition of 2.4 µl of DIEA. The reaction was quenched by addition of H_2O with 0.1% TFA, and the crude product was purified with semipreparative HPLC (gradient 20%–40% CH_3CN 0.1% TFA, 20 min, 220 nm), to afford the desired compound as an orange solid (2.3 mg, 30%). MW: 4737.6612 (neutral); Analytic HPLC (20%–50% CH_3CN 0.1% TFA, 10 min) rt 5.9 min, purity > 99%; HRMS: m/z measured 4738.2536 (2370.6268 $[M+3H]^{2+}$,1580.7501 $[M+4H]^{3+}$, 1185.8146 $[M+5H]^{4+}$, 948.8518 $[M+6H]^{5+}$)

HPLC

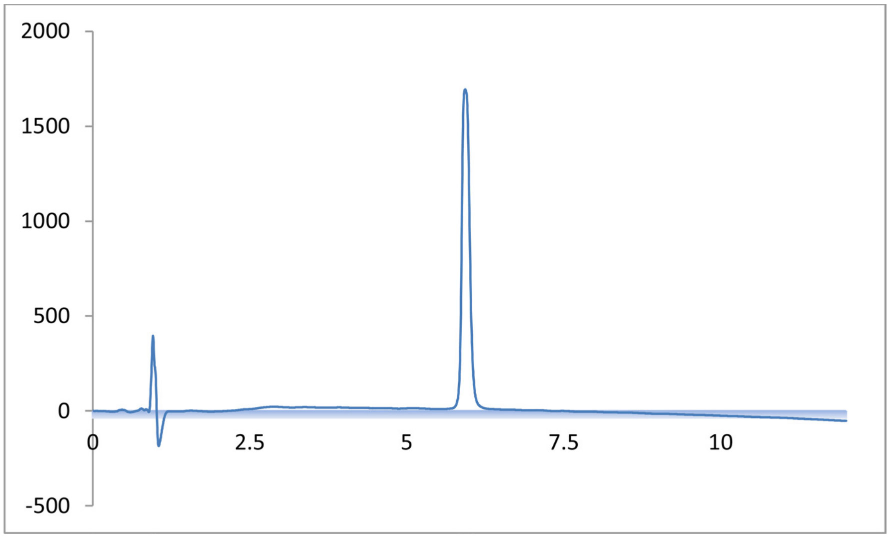

ESI

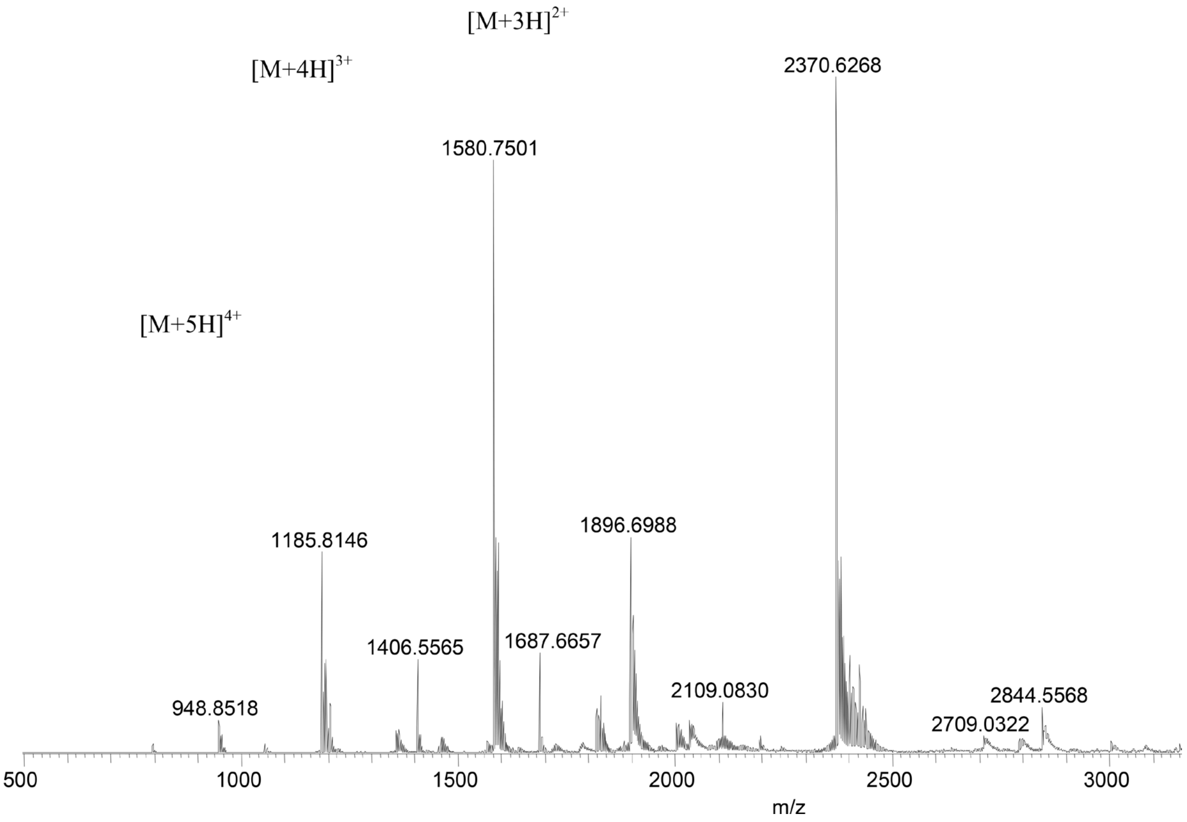

[1]Pavet V, Beyrath J, Pardin C, Morizot A, Lechner MC, Briand JP, Wendland M, Maison W, Fournel S, Micheau O, Guichard G, Gronemeyer H. Multivalent DR5 peptides activate the TRAIL death pathway and exert tumoricidal activity. Cancer Res. 2010; 70:1101–1110.
